# Bis[3-methyl-5-(pyridin-2-yl)-1*H*-pyrazol-4-yl] selenide methanol hemisolvate

**DOI:** 10.1107/S1600536813034624

**Published:** 2014-01-11

**Authors:** Maksym Seredyuk, Natalia O. Sharkina, Elzbieta Gumienna-Kontecka, Anatoly A. Kapshuk

**Affiliations:** a National Taras Shevchenko University, Department of Chemistry, Volodymyrska str. 64, 01601 Kyiv, Ukraine; b Faculty of Chemistry, University of Wroclaw, 14, F. Joliot–Curie Str., 50383, Wroclaw, Poland

## Abstract

The asymmetric unit of the title compound, C_18_H_16_N_6_Se·0.5CH_3_OH, contains two independent mol­ecules of bis­[3-methyl-5-(pyridin-2-yl)-1*H*-pyrazol-4-yl] selenide with similar C—Se—C bond angles [99.30 (14) and 98.26 (13)°], and a methanol molecule of solvation. In one mol­ecule, the dihedral angles between pyrazole and neighbouring pyridine rings are 18.3 (2) and 15.8 (2)°, and the corresponding angles in the other mol­ecule are 13.5 (2) and 8.3 (2)°. In the crystal, the selenide and solvent mol­ecules are linked by classical O—H⋯N and N—H⋯N hydrogen bonds, as well as by weak C—H⋯O and C—H⋯π inter­actions, forming a three-dimensional supra­molecular architecture.

## Related literature   

For structural studies of related pyrazol-4-ylselenides, see: Seredyuk *et al.* (2010 ▶) and for structural studies of *d*-metal complexes of pyrazol-4-ylselenide, see: Seredyuk *et al.* (2007 ▶, 2009 ▶, 2013 ▶). For related structures, see: Krämer *et al.* (2002 ▶); Penkova *et al.* (2008 ▶, 2009 ▶, 2010 ▶).
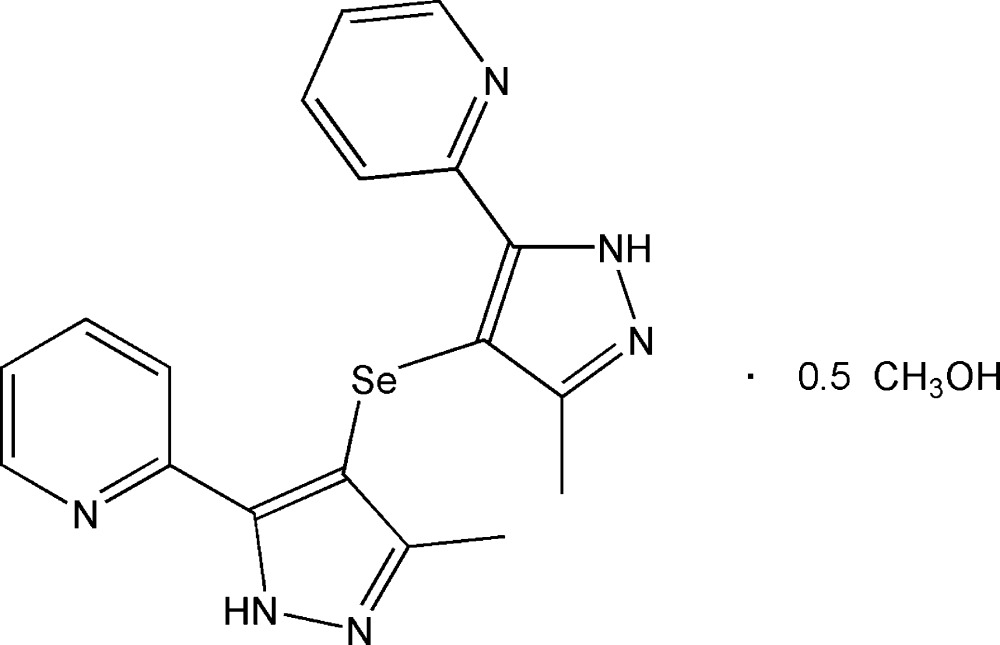


## Experimental   

### 

#### Crystal data  

2C_18_H_16_N_6_Se·CH_4_O
*M* *_r_* = 822.70
Monoclinic, 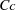

*a* = 24.386 (5) Å
*b* = 10.784 (2) Å
*c* = 15.139 (3) Å
β = 118.59 (3)°
*V* = 3495.8 (16) Å^3^
*Z* = 4
Mo *K*α radiation
μ = 2.17 mm^−1^
*T* = 120 K
0.36 × 0.24 × 0.13 mm

#### Data collection  

Bruker SMART APEXII CCD diffractometer
Absorption correction: multi-scan (*SADABS*; Bruker, 2009 ▶) *T*_min_ = 0.545, *T*_max_ = 0.767
11797 measured reflections
7632 independent reflections
6581 reflections with *I* > 2σ(*I*)
*R*_int_ = 0.054

#### Refinement  

*R*[*F* ^2^ > 2σ(*F* ^2^)] = 0.032
*wR*(*F* ^2^) = 0.078
*S* = 0.97
7632 reflections
494 parameters
2 restraints
H atoms treated by a mixture of independent and constrained refinement
Δρ_max_ = 0.62 e Å^−3^
Δρ_min_ = −0.81 e Å^−3^
Absolute structure: Flack (1983 ▶), 3629 Friedel pairs
Absolute structure parameter: −0.018 (6)

### 

Data collection: *APEX2* (Bruker, 2009 ▶); cell refinement: *SAINT* (Bruker, 2009 ▶); data reduction: *SAINT*; program(s) used to solve structure: *SIR2004* (Burla *et al.*, 2005 ▶); program(s) used to refine structure: *SHELXL97* (Sheldrick, 2008 ▶); molecular graphics: *DIAMOND* (Brandenburg, 2009 ▶); software used to prepare material for publication: *SHELXL97*.

## Supplementary Material

Crystal structure: contains datablock(s) I, New_Global_Publ_Block. DOI: 10.1107/S1600536813034624/xu5749sup1.cif

Structure factors: contains datablock(s) I. DOI: 10.1107/S1600536813034624/xu5749Isup2.hkl

Supporting information file. DOI: 10.1107/S1600536813034624/xu5749Isup3.cdx

Supporting information file. DOI: 10.1107/S1600536813034624/xu5749Isup4.cml

CCDC reference:

Additional supporting information:  crystallographic information; 3D view; checkCIF report

## Figures and Tables

**Table 1:** Hydrogen-bond geometry (Å, °) *Cg*7 is the centroid of the N21*A*-containing pyridine ring.

*D*—H⋯*A*	*D*—H	H⋯*A*	*D*⋯*A*	*D*—H⋯*A*
O1—H1*O*1⋯N4*A*	0.91 (5)	2.03 (5)	2.839 (5)	148 (5)
N1*A*—H1*A*⋯N21*A* ^i^	0.78 (4)	2.33 (4)	3.040 (4)	151 (4)
N1*B*—H1*B*⋯N21*B* ^ii^	0.73 (4)	2.32 (4)	2.988 (5)	153 (4)
N3*A*—H3*A*⋯N2*A* ^iii^	0.83 (4)	2.06 (4)	2.863 (4)	165 (4)
N3*B*—H3*B*⋯N2*B* ^iv^	0.77 (3)	2.01 (4)	2.770 (4)	170 (3)
C27*B*—H27*F*⋯O1^v^	0.96	2.32	3.273 (5)	171
C14*B*—H14*B*⋯*Cg*7^v^	0.93	2.61	3.315 (4)	133

Symmetry codes: (i) 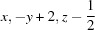; (ii) 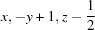; (iii) 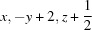; (iv) 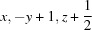; (v) 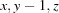.

**Fig. 1.**
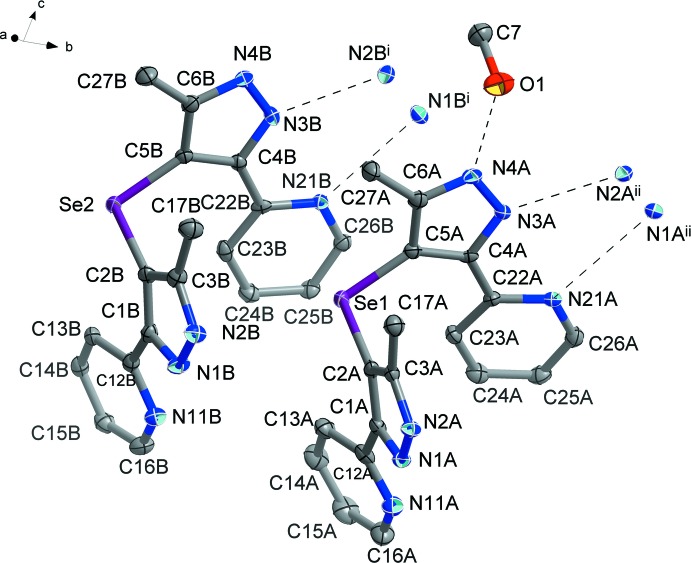
The crystal structure of the title compound showing the labeling scheme and 50% probability displacement ellipsoids. Hydrogen bonds are indicated by dashed lines. H atoms are omitted for clarity [symmetry code: (i) x, 1 - y, 0.5 + z; (ii) x, 2 - y, 0.5 + z].

**Fig. 2.**
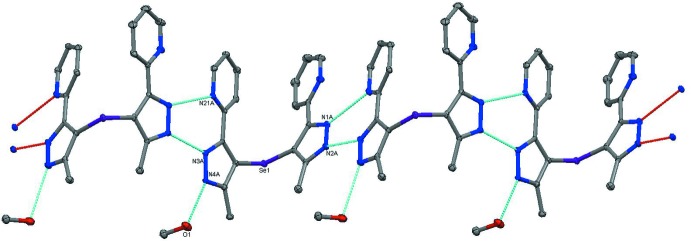
A fragment of the crystal packing showing a zigzag chain along [001] formed due to hydrogen bonding NH···N (dashed lines). Only one of the two selenide molecules is shown.

## supplementary crystallographic information

### 1. Comment

Pyrazole–derived ligands are widely used in molecular magnetism, bioinspired catalysis and supramolecular chemistry due to their bridging nature and possibility for facile functionalization with various chelating groups (Krämer *et al.*, 2002; Penkova *et al.*, 2009). As a part of our synthetic and structural study of bis(1*H*–pyrazol–4–yl)selenides (Seredyuk *et al.*, 2010) and their complexes with *d*–metals (Seredyuk *et al.*, 2007, 2009, 2013), we report here the molecular and crystal structures of the title compound (Fig. 1).

The molecule of the title compound is a symmetric organic selenide. The asymmetric unit of the title compound contains two independent molecules with the angles of C—Se—C fragments equal to 99.30 (14) and 98.26 (13)°. Additionally there is one molecule of methanol forming hydrogen bond with one of the selenide molecules [*d*(O1···N4A) = 2.839 (5) Å]. In one molecule, the dihedral angles between pyrazole and neighboring pyridine rings are 18.3 (2) and 15.8 (2)°, and the corresponding angles in another molecule are 13.5 (2) and 8.3 (2)°. The C—C, C—N, N—N bond lengths in the pyrazole ring exhibit normal values (Penkova *et al.*, 2008, 2010). The selenide molecules are united through hydrogen bonds between pyrazoles [*d*(N1A···N21A) = 2.863 (4) Å; *d*(N1B···N21B) = 2.988 (5) Å] and pyrazole and pyridine moieties [*d*(N3A···N2A) = 2.863 (4) Å; *d*(N3B···N2B) = 2.770 (4) Å] with neighboring molecules forming two different zigzag chains running along [001] (Fig. 2). Also, there are weak C—H···O and C—H···π interactions, forming the three dimensional supramolecular architecture. No π-π stacking interactions are observed between the neighboring molecules, however, there are two intermolecular contacts between pyrazole and pyridine moieties of neighbouring chains [*d*(C14B···N1A) = 3.197 (6) and *d*(C14B···C1A) = 3.393 (5) Å].

### 2. Experimental

A concentrated hot solution of bis(5-methyl-3-(pyridin-2-yl)-1*H*-pyrazol-4-yl)selenide (Seredyuk *et al.*, 2010) in methanol was cooled on air and kept overnight at ambient temperature in a sealed vial. Obtained well formed colourless crystals were filtered off and air dried. C_18.5_H_18_N_6_O_0.5_Se requires: C, 54.02; H, 4.41; N, 20.43. Found: C, 54.05; H, 4.37; N, 20.93.

### 3. Refinement

The H atoms from NH and OH were located from the difference Fourier map but were constrained to ride on their parent atom, with *U*_iso_ = 1.2*U*_eq_(parent atom) for the N—H atoms and *U*_is_ = 1.5*U*_eq_(parent atom) for the O—H atoms. The methyl and aromatic H atoms were positioned geometrically and refined as riding atoms, with C—H = 0.96 Å and *U*_iso_ = 1.5*U*_eq_(C) for the methyl H atoms and C—H = 0.93 Å and *U*_iso_ = 1.2*U*_eq_(C) for the aromatic H atoms.

### Crystal data

**Table:**

2C_18_H_16_N_6_Se·CH_4_O	*F*(000) = 1672
*M**_r_* = 822.70	*D*_x_ = 1.563 Mg m^−^^3^
Monoclinic, *C**c*	Mo *K*α radiation, λ = 0.71073 Å
Hall symbol: C -2yc	Cell parameters from 3138 reflections
*a* = 24.386 (5) Å	θ = 3.6–27.6°
*b* = 10.784 (2) Å	µ = 2.17 mm^−^^1^
*c* = 15.139 (3) Å	*T* = 120 K
β = 118.59 (3)°	Block, colorless
*V* = 3495.8 (16) Å^3^	0.36 × 0.24 × 0.13 mm
*Z* = 4	

### Data collection

**Table:**

Bruker SMART APEXII CCD diffractometer	7632 independent reflections
Radiation source: fine-focus sealed tube	6581 reflections with *I* > 2σ(*I*)
Horizontally mounted graphite crystal monochromator	*R*_int_ = 0.054
Detector resolution: 9 pixels mm^-1^	θ_max_ = 28.5°, θ_min_ = 3.6°
φ scans and ω scans with κ offset	*h* = −32→32
Absorption correction: multi-scan (*SADABS*; Bruker, 2009)	*k* = −8→14
*T*_min_ = 0.545, *T*_max_ = 0.767	*l* = −19→19
11797 measured reflections	

### Refinement

**Table:**

Refinement on *F*^2^	Secondary atom site location: difference Fourier map
Least-squares matrix: full	Hydrogen site location: inferred from neighbouring sites
*R*[*F*^2^ > 2σ(*F*^2^)] = 0.032	H atoms treated by a mixture of independent and constrained refinement
*wR*(*F*^2^) = 0.078	*w* = 1/[σ^2^(*F*_o_^2^) + (0.0407*P*)^2^] where *P* = (*F*_o_^2^ + 2*F*_c_^2^)/3
*S* = 0.97	(Δ/σ)_max_ = 0.001
7632 reflections	Δρ_max_ = 0.62 e Å^−^^3^
494 parameters	Δρ_min_ = −0.81 e Å^−^^3^
2 restraints	Absolute structure: Flack (1983), 3629 Friedel pairs
Primary atom site location: structure-invariant direct methods	Absolute structure parameter: −0.018 (6)

### Special details

**Table:**

Experimental. The H atoms from NH and OH were located from the difference Fourier map but were constrained to ride on their parent atom, with *U*_iso_ = 1.2*U*_eq_(parent atom) for the N—H atoms and *U*_is_ = 1.5*U*_eq_(parent atom) for the O—H atoms. The methyl and aromatic H atoms were positioned geometrically and refined as riding atoms, with C—H = 0.96 Å and *U*_iso_ = 1.5*U*_eq_(C) for the methyl H atoms and C—H = 0.93 Å and *U*_iso_ = 1.2*U*_eq_(C) for the aromatic H atoms.
Geometry. All e.s.d.'s (except the e.s.d. in the dihedral angle between two l.s. planes) are estimated using the full covariance matrix. The cell e.s.d.'s are taken into account individually in the estimation of e.s.d.'s in distances, angles and torsion angles; correlations between e.s.d.'s in cell parameters are only used when they are defined by crystal symmetry. An approximate (isotropic) treatment of cell e.s.d.'s is used for estimating e.s.d.'s involving l.s. planes.
Refinement. Refinement of *F*^2^ against ALL reflections. The weighted *R*-factor *wR* and goodness of fit *S* are based on *F*^2^, conventional *R*-factors *R* are based on *F*, with *F* set to zero for negative *F*^2^. The threshold expression of *F*^2^ > σ(*F*^2^) is used only for calculating *R*-factors(gt) *etc*. and is not relevant to the choice of reflections for refinement. *R*-factors based on *F*^2^ are statistically about twice as large as those based on *F*, and *R*- factors based on ALL data will be even larger.

### Fractional atomic coordinates and isotropic or equivalent isotropic displacement parameters (Å^2^)

**Table:**

	*x*	*y*	*z*	*U*_iso_*/*U*_eq_	
Se1	0.355504 (12)	0.63083 (3)	0.018781 (17)	0.01693 (11)	
Se2	0.429962 (12)	0.13792 (3)	0.106831 (16)	0.01682 (11)	
O1	0.54984 (14)	0.9478 (3)	0.4101 (2)	0.0366 (7)	
N1A	0.29572 (16)	0.8389 (3)	−0.2336 (3)	0.0171 (7)	
N1B	0.36479 (18)	0.3522 (3)	−0.1434 (3)	0.0219 (9)	
N2A	0.35458 (15)	0.8832 (3)	−0.1848 (3)	0.0175 (7)	
N2B	0.42002 (16)	0.4077 (3)	−0.0902 (3)	0.0208 (7)	
N3A	0.37107 (15)	0.9089 (3)	0.2126 (2)	0.0168 (7)	
N3B	0.43950 (16)	0.4036 (3)	0.3037 (3)	0.0185 (7)	
N4A	0.43116 (16)	0.8668 (3)	0.2581 (3)	0.0199 (8)	
N4B	0.50018 (16)	0.3670 (3)	0.3518 (3)	0.0200 (8)	
N11A	0.17652 (13)	0.7566 (3)	−0.3020 (2)	0.0240 (6)	
N11B	0.25090 (12)	0.2489 (3)	−0.2249 (2)	0.0216 (6)	
N21A	0.24727 (12)	0.9800 (2)	0.09135 (19)	0.0177 (5)	
N21B	0.31604 (12)	0.4718 (2)	0.18315 (19)	0.0158 (5)	
C1A	0.28369 (17)	0.7508 (3)	−0.1820 (3)	0.0154 (7)	
C1B	0.35530 (18)	0.2556 (3)	−0.0949 (3)	0.0168 (7)	
C2A	0.33882 (17)	0.7373 (3)	−0.0920 (3)	0.0166 (8)	
C2B	0.40936 (18)	0.2493 (3)	−0.0023 (3)	0.0169 (8)	
C3A	0.38170 (18)	0.8204 (3)	−0.0981 (3)	0.0155 (7)	
C3B	0.4480 (2)	0.3469 (4)	−0.0026 (3)	0.0193 (9)	
C4A	0.33359 (19)	0.8424 (4)	0.1301 (3)	0.0136 (7)	
C4B	0.40427 (18)	0.3416 (3)	0.2179 (3)	0.0146 (7)	
C5A	0.37217 (17)	0.7535 (3)	0.1200 (3)	0.0162 (7)	
C5B	0.44480 (17)	0.2577 (3)	0.2088 (3)	0.0154 (7)	
C6A	0.43134 (18)	0.7730 (3)	0.2017 (3)	0.0189 (8)	
C6B	0.50331 (17)	0.2784 (3)	0.2931 (3)	0.0200 (8)	
C7	0.55599 (18)	0.8730 (3)	0.4891 (3)	0.0303 (8)	
H7A	0.5975	0.8808	0.5444	0.045*	
H7B	0.5484	0.7882	0.4673	0.045*	
H7C	0.5263	0.8983	0.5102	0.045*	
C12A	0.22154 (16)	0.6938 (3)	−0.2267 (3)	0.0184 (7)	
C12B	0.29543 (16)	0.1904 (3)	−0.1438 (3)	0.0181 (7)	
C13A	0.21192 (17)	0.5802 (3)	−0.1921 (3)	0.0245 (7)	
H13A	0.2448	0.5369	−0.1411	0.029*	
C13B	0.28421 (15)	0.0781 (3)	−0.1087 (2)	0.0174 (6)	
H13B	0.3159	0.0389	−0.0529	0.021*	
C14A	0.15067 (17)	0.5340 (3)	−0.2372 (3)	0.0303 (8)	
H14A	0.1420	0.4595	−0.2154	0.036*	
C14B	0.22572 (15)	0.0272 (3)	−0.1579 (2)	0.0195 (7)	
H14B	0.2173	−0.0468	−0.1353	0.023*	
C15A	0.10368 (18)	0.5995 (4)	−0.3138 (3)	0.0344 (9)	
H15A	0.0626	0.5715	−0.3438	0.041*	
C15B	0.17929 (16)	0.0859 (3)	−0.2412 (3)	0.0232 (7)	
H15B	0.1392	0.0530	−0.2754	0.028*	
C16A	0.11907 (17)	0.7087 (3)	−0.3452 (3)	0.0326 (9)	
H16A	0.0876	0.7508	−0.3993	0.039*	
C16B	0.19462 (16)	0.1959 (3)	−0.2721 (3)	0.0256 (7)	
H16B	0.1638	0.2349	−0.3290	0.031*	
C17A	0.4486 (2)	0.8426 (4)	−0.0230 (4)	0.0239 (9)	
H17A	0.4662	0.9025	−0.0492	0.036*	
H17B	0.4714	0.7662	−0.0099	0.036*	
H17C	0.4512	0.8733	0.0384	0.036*	
C17B	0.51041 (19)	0.3890 (4)	0.0777 (3)	0.0267 (9)	
H17D	0.5278	0.4470	0.0498	0.040*	
H17E	0.5377	0.3188	0.1042	0.040*	
H17F	0.5058	0.4283	0.1307	0.040*	
C22A	0.26647 (16)	0.8694 (3)	0.0726 (3)	0.0146 (7)	
C22B	0.33795 (19)	0.3707 (3)	0.1561 (3)	0.0137 (8)	
C23A	0.22481 (15)	0.7835 (3)	0.0046 (2)	0.0182 (6)	
H23A	0.2393	0.7077	−0.0054	0.022*	
C23B	0.29889 (14)	0.2977 (3)	0.0742 (2)	0.0179 (6)	
H23B	0.3145	0.2273	0.0585	0.021*	
C24A	0.16218 (15)	0.8117 (3)	−0.0476 (3)	0.0226 (7)	
H24A	0.1340	0.7558	−0.0937	0.027*	
C24B	0.23644 (16)	0.3303 (3)	0.0160 (3)	0.0215 (7)	
H24B	0.2102	0.2832	−0.0399	0.026*	
C25A	0.14153 (15)	0.9257 (3)	−0.0306 (2)	0.0231 (7)	
H25A	0.0996	0.9476	−0.0650	0.028*	
C25B	0.21373 (14)	0.4343 (3)	0.0424 (2)	0.0188 (7)	
H25B	0.1723	0.4589	0.0049	0.023*	
C26A	0.18575 (15)	1.0048 (3)	0.0394 (2)	0.0208 (7)	
H26A	0.1720	1.0802	0.0513	0.025*	
C26B	0.25535 (14)	0.4998 (3)	0.1269 (2)	0.0188 (6)	
H26B	0.2401	0.5680	0.1459	0.023*	
C27A	0.49055 (17)	0.7012 (3)	0.2310 (3)	0.0237 (8)	
H27A	0.5259	0.7535	0.2697	0.036*	
H27B	0.4916	0.6740	0.1714	0.036*	
H27C	0.4919	0.6305	0.2704	0.036*	
C27B	0.56453 (17)	0.2152 (4)	0.3213 (3)	0.0256 (9)	
H27D	0.5982	0.2650	0.3696	0.038*	
H27E	0.5693	0.2049	0.2624	0.038*	
H27F	0.5652	0.1354	0.3500	0.038*	
H1A	0.2724 (18)	0.868 (3)	−0.285 (3)	0.018 (10)*	
H1B	0.3444 (19)	0.377 (3)	−0.193 (3)	0.021 (11)*	
H3A	0.3594 (18)	0.969 (4)	0.234 (3)	0.032 (11)*	
H3B	0.4296 (14)	0.455 (3)	0.329 (2)	0.007 (8)*	
H1O1	0.508 (2)	0.943 (5)	0.375 (4)	0.068 (17)*	

### Atomic displacement parameters (Å^2^)

**Table:**

	*U*^11^	*U*^22^	*U*^33^	*U*^12^	*U*^13^	*U*^23^
Se1	0.0222 (2)	0.01311 (19)	0.0151 (2)	0.00129 (14)	0.0086 (2)	0.00062 (14)
Se2	0.0222 (2)	0.01314 (19)	0.0158 (2)	0.00342 (15)	0.0097 (2)	0.00146 (15)
O1	0.0315 (15)	0.0364 (15)	0.0316 (14)	−0.0089 (12)	0.0068 (13)	0.0070 (12)
N1A	0.0182 (15)	0.0185 (15)	0.0125 (14)	0.0000 (12)	0.0058 (13)	0.0008 (12)
N1B	0.0228 (19)	0.0223 (17)	0.0177 (18)	−0.0013 (13)	0.0073 (16)	0.0057 (13)
N2A	0.0160 (15)	0.0192 (15)	0.0149 (15)	−0.0034 (11)	0.0056 (13)	−0.0006 (12)
N2B	0.0212 (17)	0.0208 (17)	0.0187 (17)	−0.0070 (14)	0.0082 (15)	−0.0001 (14)
N3A	0.0173 (16)	0.0173 (15)	0.0128 (15)	0.0033 (13)	0.0047 (14)	−0.0010 (13)
N3B	0.0222 (16)	0.0171 (15)	0.0179 (15)	0.0008 (13)	0.0110 (14)	−0.0043 (13)
N4A	0.0130 (15)	0.0256 (17)	0.0146 (15)	0.0047 (12)	0.0013 (13)	0.0010 (12)
N4B	0.0138 (15)	0.0221 (16)	0.0169 (16)	0.0048 (12)	0.0015 (13)	−0.0003 (12)
N11A	0.0199 (14)	0.0250 (15)	0.0224 (15)	0.0004 (11)	0.0065 (13)	−0.0011 (12)
N11B	0.0213 (15)	0.0240 (15)	0.0188 (14)	0.0006 (11)	0.0089 (13)	0.0029 (12)
N21A	0.0185 (13)	0.0191 (13)	0.0119 (12)	0.0011 (10)	0.0045 (11)	0.0007 (10)
N21B	0.0214 (13)	0.0143 (12)	0.0138 (12)	0.0010 (10)	0.0100 (12)	0.0005 (10)
C1A	0.0183 (16)	0.0146 (15)	0.0143 (15)	−0.0028 (12)	0.0087 (14)	−0.0039 (13)
C1B	0.0195 (17)	0.0137 (15)	0.0191 (17)	0.0009 (12)	0.0107 (15)	0.0020 (13)
C2A	0.024 (2)	0.0120 (15)	0.0171 (17)	0.0008 (13)	0.0131 (17)	−0.0005 (13)
C2B	0.023 (2)	0.0155 (17)	0.0156 (18)	−0.0009 (14)	0.0122 (17)	0.0007 (14)
C3A	0.0185 (19)	0.0126 (16)	0.0148 (17)	−0.0009 (14)	0.0075 (16)	−0.0025 (14)
C3B	0.020 (2)	0.0191 (19)	0.021 (2)	0.0001 (16)	0.0119 (19)	0.0018 (16)
C4A	0.0144 (18)	0.0171 (16)	0.0092 (17)	0.0000 (14)	0.0055 (16)	0.0039 (14)
C4B	0.0177 (18)	0.0142 (16)	0.0145 (17)	0.0002 (13)	0.0098 (16)	0.0008 (13)
C5A	0.0171 (18)	0.0160 (16)	0.0135 (16)	−0.0015 (14)	0.0056 (16)	0.0009 (14)
C5B	0.0181 (17)	0.0148 (16)	0.0132 (15)	0.0013 (13)	0.0074 (15)	0.0018 (13)
C6A	0.0216 (18)	0.0191 (17)	0.0176 (17)	0.0011 (13)	0.0106 (15)	0.0025 (13)
C6B	0.0217 (18)	0.0202 (17)	0.0188 (17)	0.0050 (14)	0.0102 (16)	0.0036 (14)
C7	0.033 (2)	0.029 (2)	0.0290 (18)	0.0012 (16)	0.0145 (18)	0.0032 (16)
C12A	0.0210 (17)	0.0202 (17)	0.0173 (17)	−0.0065 (13)	0.0118 (15)	−0.0065 (14)
C12B	0.0211 (17)	0.0151 (15)	0.0205 (17)	0.0021 (13)	0.0120 (15)	−0.0001 (14)
C13A	0.0258 (17)	0.0222 (18)	0.0203 (16)	−0.0049 (15)	0.0068 (15)	−0.0041 (15)
C13B	0.0220 (16)	0.0131 (15)	0.0162 (15)	0.0008 (13)	0.0085 (14)	−0.0014 (12)
C14A	0.0307 (19)	0.0266 (19)	0.033 (2)	−0.0112 (15)	0.0143 (18)	−0.0045 (16)
C14B	0.0250 (17)	0.0160 (15)	0.0219 (16)	−0.0015 (13)	0.0147 (15)	−0.0020 (13)
C15A	0.0237 (19)	0.035 (2)	0.040 (2)	−0.0127 (16)	0.0119 (18)	−0.0100 (18)
C15B	0.0199 (17)	0.0254 (17)	0.0223 (17)	−0.0027 (14)	0.0084 (15)	−0.0047 (14)
C16A	0.0221 (18)	0.030 (2)	0.033 (2)	0.0001 (15)	0.0029 (17)	−0.0064 (17)
C16B	0.0227 (17)	0.0296 (19)	0.0188 (16)	0.0022 (15)	0.0053 (15)	0.0038 (15)
C17A	0.025 (2)	0.0212 (19)	0.023 (2)	−0.0025 (16)	0.010 (2)	−0.0007 (16)
C17B	0.027 (2)	0.0283 (19)	0.0236 (19)	−0.0030 (16)	0.0115 (17)	0.0012 (16)
C22A	0.0142 (15)	0.0148 (16)	0.0120 (16)	0.0015 (12)	0.0042 (14)	0.0012 (12)
C22B	0.0130 (18)	0.0184 (18)	0.0117 (17)	−0.0012 (13)	0.0075 (16)	0.0041 (13)
C23A	0.0212 (16)	0.0156 (15)	0.0162 (14)	−0.0019 (12)	0.0076 (14)	−0.0031 (12)
C23B	0.0230 (16)	0.0156 (15)	0.0208 (16)	−0.0011 (12)	0.0152 (15)	−0.0022 (13)
C24A	0.0212 (17)	0.0233 (17)	0.0212 (17)	−0.0073 (14)	0.0085 (15)	−0.0009 (14)
C24B	0.0214 (17)	0.0221 (17)	0.0192 (16)	−0.0039 (13)	0.0082 (15)	−0.0027 (14)
C25A	0.0164 (16)	0.0296 (18)	0.0206 (16)	0.0041 (14)	0.0066 (14)	0.0037 (14)
C25B	0.0127 (15)	0.0224 (16)	0.0200 (16)	0.0006 (12)	0.0068 (14)	0.0004 (13)
C26A	0.0224 (17)	0.0191 (16)	0.0227 (17)	0.0038 (13)	0.0122 (15)	0.0013 (13)
C26B	0.0210 (16)	0.0177 (15)	0.0178 (15)	0.0033 (12)	0.0095 (14)	0.0036 (13)
C27A	0.0207 (18)	0.0246 (18)	0.0220 (19)	0.0055 (14)	0.0072 (16)	−0.0035 (15)
C27B	0.0188 (18)	0.029 (2)	0.023 (2)	0.0044 (15)	0.0054 (16)	0.0000 (16)

### Geometric parameters (Å, º)

**Table:**

Se1—C2A	1.908 (4)	C7—H7B	0.9600
Se1—C5A	1.915 (4)	C7—H7C	0.9600
Se2—C2B	1.905 (4)	C12A—C13A	1.396 (5)
Se2—C5B	1.910 (4)	C12B—C13B	1.401 (5)
O1—C7	1.390 (4)	C13A—C14A	1.404 (5)
O1—H1O1	0.91 (5)	C13A—H13A	0.9300
N1A—C1A	1.347 (5)	C13B—C14B	1.369 (4)
N1A—N2A	1.349 (5)	C13B—H13B	0.9300
N1A—H1A	0.78 (4)	C14A—C15A	1.373 (6)
N1B—N2B	1.335 (5)	C14A—H14A	0.9300
N1B—C1B	1.356 (5)	C14B—C15B	1.380 (5)
N1B—H1B	0.73 (4)	C14B—H14B	0.9300
N2A—C3A	1.337 (5)	C15A—C16A	1.387 (5)
N2B—C3B	1.337 (5)	C15A—H15A	0.9300
N3A—C4A	1.348 (5)	C15B—C16B	1.390 (5)
N3A—N4A	1.365 (5)	C15B—H15B	0.9300
N3A—H3A	0.83 (4)	C16A—H16A	0.9300
N3B—C4B	1.343 (5)	C16B—H16B	0.9300
N3B—N4B	1.358 (5)	C17A—H17A	0.9600
N3B—H3B	0.77 (3)	C17A—H17B	0.9600
N4A—C6A	1.325 (5)	C17A—H17C	0.9600
N4B—C6B	1.332 (5)	C17B—H17D	0.9600
N11A—C12A	1.328 (4)	C17B—H17E	0.9600
N11A—C16A	1.334 (4)	C17B—H17F	0.9600
N11B—C16B	1.335 (4)	C22A—C23A	1.397 (4)
N11B—C12B	1.345 (4)	C22B—C23B	1.390 (5)
N21A—C26A	1.346 (4)	C23A—C24A	1.377 (5)
N21A—C22A	1.359 (4)	C23A—H23A	0.9300
N21B—C26B	1.342 (4)	C23B—C24B	1.391 (5)
N21B—C22B	1.361 (4)	C23B—H23B	0.9300
C1A—C2A	1.391 (5)	C24A—C25A	1.398 (5)
C1A—C12A	1.467 (5)	C24A—H24A	0.9300
C1B—C2B	1.393 (5)	C24B—C25B	1.392 (4)
C1B—C12B	1.462 (5)	C24B—H24B	0.9300
C2A—C3A	1.413 (5)	C25A—C26A	1.386 (5)
C2B—C3B	1.414 (5)	C25A—H25A	0.9300
C3A—C17A	1.496 (6)	C25B—C26B	1.387 (4)
C3B—C17B	1.494 (6)	C25B—H25B	0.9300
C4A—C5A	1.402 (5)	C26A—H26A	0.9300
C4A—C22A	1.469 (5)	C26B—H26B	0.9300
C4B—C5B	1.395 (5)	C27A—H27A	0.9600
C4B—C22B	1.464 (6)	C27A—H27B	0.9600
C5A—C6A	1.396 (5)	C27A—H27C	0.9600
C5B—C6B	1.404 (5)	C27B—H27D	0.9600
C6A—C27A	1.506 (5)	C27B—H27E	0.9600
C6B—C27B	1.505 (5)	C27B—H27F	0.9600
C7—H7A	0.9600		
			
C2A—Se1—C5A	99.30 (14)	C14B—C13B—H13B	120.6
C2B—Se2—C5B	98.26 (13)	C12B—C13B—H13B	120.6
C7—O1—H1O1	96 (3)	C15A—C14A—C13A	119.5 (3)
C1A—N1A—N2A	113.7 (3)	C15A—C14A—H14A	120.3
C1A—N1A—H1A	128 (3)	C13A—C14A—H14A	120.3
N2A—N1A—H1A	119 (3)	C13B—C14B—C15B	119.9 (3)
N2B—N1B—C1B	113.7 (4)	C13B—C14B—H14B	120.0
N2B—N1B—H1B	117 (3)	C15B—C14B—H14B	120.0
C1B—N1B—H1B	130 (3)	C14A—C15A—C16A	118.2 (3)
C3A—N2A—N1A	104.8 (3)	C14A—C15A—H15A	120.9
N1B—N2B—C3B	105.5 (3)	C16A—C15A—H15A	120.9
C4A—N3A—N4A	112.2 (3)	C14B—C15B—C16B	117.6 (3)
C4A—N3A—H3A	124 (3)	C14B—C15B—H15B	121.2
N4A—N3A—H3A	123 (3)	C16B—C15B—H15B	121.2
C4B—N3B—N4B	113.7 (3)	N11A—C16A—C15A	123.6 (3)
C4B—N3B—H3B	129 (2)	N11A—C16A—H16A	118.2
N4B—N3B—H3B	117 (2)	C15A—C16A—H16A	118.2
C6A—N4A—N3A	105.2 (3)	N11B—C16B—C15B	124.0 (3)
C6B—N4B—N3B	104.3 (3)	N11B—C16B—H16B	118.0
C12A—N11A—C16A	117.8 (3)	C15B—C16B—H16B	118.0
C16B—N11B—C12B	117.5 (3)	C3A—C17A—H17A	109.5
C26A—N21A—C22A	116.8 (3)	C3A—C17A—H17B	109.5
C26B—N21B—C22B	117.5 (3)	H17A—C17A—H17B	109.5
N1A—C1A—C2A	105.4 (3)	C3A—C17A—H17C	109.5
N1A—C1A—C12A	119.5 (3)	H17A—C17A—H17C	109.5
C2A—C1A—C12A	135.2 (3)	H17B—C17A—H17C	109.5
N1B—C1B—C2B	104.9 (3)	C3B—C17B—H17D	109.5
N1B—C1B—C12B	118.9 (3)	C3B—C17B—H17E	109.5
C2B—C1B—C12B	136.1 (3)	H17D—C17B—H17E	109.5
C1A—C2A—C3A	105.5 (3)	C3B—C17B—H17F	109.5
C1A—C2A—Se1	128.6 (3)	H17D—C17B—H17F	109.5
C3A—C2A—Se1	125.8 (3)	H17E—C17B—H17F	109.5
C1B—C2B—C3B	105.8 (3)	N21A—C22A—C23A	122.3 (3)
C1B—C2B—Se2	129.3 (3)	N21A—C22A—C4A	116.6 (3)
C3B—C2B—Se2	124.9 (3)	C23A—C22A—C4A	121.1 (3)
N2A—C3A—C2A	110.6 (3)	N21B—C22B—C23B	121.5 (3)
N2A—C3A—C17A	120.8 (4)	N21B—C22B—C4B	116.8 (3)
C2A—C3A—C17A	128.6 (4)	C23B—C22B—C4B	121.7 (3)
N2B—C3B—C2B	110.1 (4)	C24A—C23A—C22A	119.5 (3)
N2B—C3B—C17B	119.7 (4)	C24A—C23A—H23A	120.3
C2B—C3B—C17B	130.1 (4)	C22A—C23A—H23A	120.3
N3A—C4A—C5A	105.8 (3)	C22B—C23B—C24B	119.8 (3)
N3A—C4A—C22A	120.8 (4)	C22B—C23B—H23B	120.1
C5A—C4A—C22A	133.3 (4)	C24B—C23B—H23B	120.1
N3B—C4B—C5B	105.3 (3)	C23A—C24A—C25A	119.2 (3)
N3B—C4B—C22B	120.8 (3)	C23A—C24A—H24A	120.4
C5B—C4B—C22B	133.9 (4)	C25A—C24A—H24A	120.4
C6A—C5A—C4A	105.3 (3)	C23B—C24B—C25B	119.1 (3)
C6A—C5A—Se1	123.1 (3)	C23B—C24B—H24B	120.4
C4A—C5A—Se1	131.5 (3)	C25B—C24B—H24B	120.4
C4B—C5B—C6B	105.5 (3)	C26A—C25A—C24A	117.7 (3)
C4B—C5B—Se2	130.5 (3)	C26A—C25A—H25A	121.1
C6B—C5B—Se2	124.0 (3)	C24A—C25A—H25A	121.1
N4A—C6A—C5A	111.4 (3)	C26B—C25B—C24B	117.4 (3)
N4A—C6A—C27A	120.1 (3)	C26B—C25B—H25B	121.3
C5A—C6A—C27A	128.5 (3)	C24B—C25B—H25B	121.3
N4B—C6B—C5B	111.3 (3)	N21A—C26A—C25A	124.5 (3)
N4B—C6B—C27B	120.1 (3)	N21A—C26A—H26A	117.8
C5B—C6B—C27B	128.6 (3)	C25A—C26A—H26A	117.8
O1—C7—H7A	109.5	N21B—C26B—C25B	124.7 (3)
O1—C7—H7B	109.5	N21B—C26B—H26B	117.7
H7A—C7—H7B	109.5	C25B—C26B—H26B	117.7
O1—C7—H7C	109.5	C6A—C27A—H27A	109.5
H7A—C7—H7C	109.5	C6A—C27A—H27B	109.5
H7B—C7—H7C	109.5	H27A—C27A—H27B	109.5
N11A—C12A—C13A	123.4 (3)	C6A—C27A—H27C	109.5
N11A—C12A—C1A	115.4 (3)	H27A—C27A—H27C	109.5
C13A—C12A—C1A	121.1 (3)	H27B—C27A—H27C	109.5
N11B—C12B—C13B	122.2 (3)	C6B—C27B—H27D	109.5
N11B—C12B—C1B	114.2 (3)	C6B—C27B—H27E	109.5
C13B—C12B—C1B	123.5 (3)	H27D—C27B—H27E	109.5
C12A—C13A—C14A	117.4 (3)	C6B—C27B—H27F	109.5
C12A—C13A—H13A	121.3	H27D—C27B—H27F	109.5
C14A—C13A—H13A	121.3	H27E—C27B—H27F	109.5
C14B—C13B—C12B	118.8 (3)		
			
C1A—N1A—N2A—C3A	0.3 (4)	N3B—N4B—C6B—C5B	−0.7 (4)
C1B—N1B—N2B—C3B	−0.9 (5)	N3B—N4B—C6B—C27B	179.1 (3)
C4A—N3A—N4A—C6A	0.9 (4)	C4B—C5B—C6B—N4B	0.8 (4)
C4B—N3B—N4B—C6B	0.5 (4)	Se2—C5B—C6B—N4B	−179.9 (3)
N2A—N1A—C1A—C2A	0.6 (4)	C4B—C5B—C6B—C27B	−179.1 (4)
N2A—N1A—C1A—C12A	−179.6 (3)	Se2—C5B—C6B—C27B	0.2 (5)
N2B—N1B—C1B—C2B	0.3 (5)	C16A—N11A—C12A—C13A	1.1 (5)
N2B—N1B—C1B—C12B	177.5 (3)	C16A—N11A—C12A—C1A	−180.0 (3)
N1A—C1A—C2A—C3A	−1.2 (4)	N1A—C1A—C12A—N11A	−18.1 (5)
C12A—C1A—C2A—C3A	179.0 (4)	C2A—C1A—C12A—N11A	161.6 (4)
N1A—C1A—C2A—Se1	178.8 (3)	N1A—C1A—C12A—C13A	160.8 (3)
C12A—C1A—C2A—Se1	−1.0 (6)	C2A—C1A—C12A—C13A	−19.4 (6)
C5A—Se1—C2A—C1A	−116.8 (3)	C16B—N11B—C12B—C13B	−0.1 (5)
C5A—Se1—C2A—C3A	63.2 (3)	C16B—N11B—C12B—C1B	−178.7 (3)
N1B—C1B—C2B—C3B	0.4 (4)	N1B—C1B—C12B—N11B	−12.2 (5)
C12B—C1B—C2B—C3B	−176.1 (4)	C2B—C1B—C12B—N11B	164.0 (4)
N1B—C1B—C2B—Se2	179.3 (3)	N1B—C1B—C12B—C13B	169.3 (3)
C12B—C1B—C2B—Se2	2.8 (7)	C2B—C1B—C12B—C13B	−14.5 (7)
C5B—Se2—C2B—C1B	−122.5 (4)	N11A—C12A—C13A—C14A	−2.8 (5)
C5B—Se2—C2B—C3B	56.2 (4)	C1A—C12A—C13A—C14A	178.3 (3)
N1A—N2A—C3A—C2A	−1.1 (4)	N11B—C12B—C13B—C14B	−0.9 (5)
N1A—N2A—C3A—C17A	179.1 (3)	C1B—C12B—C13B—C14B	177.6 (3)
C1A—C2A—C3A—N2A	1.5 (4)	C12A—C13A—C14A—C15A	1.4 (5)
Se1—C2A—C3A—N2A	−178.5 (3)	C12B—C13B—C14B—C15B	0.7 (5)
C1A—C2A—C3A—C17A	−178.7 (4)	C13A—C14A—C15A—C16A	1.4 (6)
Se1—C2A—C3A—C17A	1.3 (6)	C13B—C14B—C15B—C16B	0.4 (5)
N1B—N2B—C3B—C2B	1.1 (5)	C12A—N11A—C16A—C15A	2.0 (5)
N1B—N2B—C3B—C17B	−177.3 (4)	C14A—C15A—C16A—N11A	−3.3 (6)
C1B—C2B—C3B—N2B	−1.0 (5)	C12B—N11B—C16B—C15B	1.3 (5)
Se2—C2B—C3B—N2B	−180.0 (3)	C14B—C15B—C16B—N11B	−1.4 (5)
C1B—C2B—C3B—C17B	177.2 (4)	C26A—N21A—C22A—C23A	−1.4 (5)
Se2—C2B—C3B—C17B	−1.8 (6)	C26A—N21A—C22A—C4A	−179.5 (3)
N4A—N3A—C4A—C5A	−1.5 (4)	N3A—C4A—C22A—N21A	15.2 (5)
N4A—N3A—C4A—C22A	177.4 (3)	C5A—C4A—C22A—N21A	−166.2 (4)
N4B—N3B—C4B—C5B	0.0 (4)	N3A—C4A—C22A—C23A	−162.9 (3)
N4B—N3B—C4B—C22B	−179.2 (3)	C5A—C4A—C22A—C23A	15.6 (6)
N3A—C4A—C5A—C6A	1.4 (4)	C26B—N21B—C22B—C23B	−1.0 (5)
C22A—C4A—C5A—C6A	−177.3 (4)	C26B—N21B—C22B—C4B	179.8 (3)
N3A—C4A—C5A—Se1	−176.2 (3)	N3B—C4B—C22B—N21B	7.5 (5)
C22A—C4A—C5A—Se1	5.1 (7)	C5B—C4B—C22B—N21B	−171.4 (4)
C2A—Se1—C5A—C6A	−110.9 (3)	N3B—C4B—C22B—C23B	−171.6 (4)
C2A—Se1—C5A—C4A	66.4 (4)	C5B—C4B—C22B—C23B	9.5 (7)
N3B—C4B—C5B—C6B	−0.4 (4)	N21A—C22A—C23A—C24A	1.6 (5)
C22B—C4B—C5B—C6B	178.6 (4)	C4A—C22A—C23A—C24A	179.7 (3)
N3B—C4B—C5B—Se2	−179.7 (3)	N21B—C22B—C23B—C24B	2.4 (5)
C22B—C4B—C5B—Se2	−0.7 (7)	C4B—C22B—C23B—C24B	−178.5 (3)
C2B—Se2—C5B—C4B	64.7 (4)	C22A—C23A—C24A—C25A	−0.7 (5)
C2B—Se2—C5B—C6B	−114.4 (3)	C22B—C23B—C24B—C25B	−1.6 (5)
N3A—N4A—C6A—C5A	0.0 (4)	C23A—C24A—C25A—C26A	−0.4 (5)
N3A—N4A—C6A—C27A	−178.6 (3)	C23B—C24B—C25B—C26B	−0.3 (5)
C4A—C5A—C6A—N4A	−0.9 (4)	C22A—N21A—C26A—C25A	0.2 (5)
Se1—C5A—C6A—N4A	177.0 (3)	C24A—C25A—C26A—N21A	0.7 (5)
C4A—C5A—C6A—C27A	177.6 (4)	C22B—N21B—C26B—C25B	−1.1 (5)
Se1—C5A—C6A—C27A	−4.5 (5)	C24B—C25B—C26B—N21B	1.8 (5)

### Hydrogen-bond geometry (Å, º)

Cg7 is the centroid of the N21A-containing pyridine ring.

**Table:**

*D*—H···*A*	*D*—H	H···*A*	*D*···*A*	*D*—H···*A*
O1—H1*O*1···N4*A*	0.91 (5)	2.03 (5)	2.839 (5)	148 (5)
N1*A*—H1*A*···N21*A*^i^	0.78 (4)	2.33 (4)	3.040 (4)	151 (4)
N1*B*—H1*B*···N21*B*^ii^	0.73 (4)	2.32 (4)	2.988 (5)	153 (4)
N3*A*—H3*A*···N2*A*^iii^	0.83 (4)	2.06 (4)	2.863 (4)	165 (4)
N3*B*—H3*B*···N2*B*^iv^	0.77 (3)	2.01 (4)	2.770 (4)	170 (3)
C27*B*—H27*F*···O1^v^	0.96	2.32	3.273 (5)	171
C14*B*—H14*B*···*Cg*7^v^	0.93	2.61	3.315 (4)	133

Symmetry codes: (i) *x*, −*y*+2, *z*−1/2; (ii) *x*, −*y*+1, *z*−1/2; (iii) *x*, −*y*+2, *z*+1/2; (iv) *x*, −*y*+1, *z*+1/2; (v) *x*, *y*−1, *z*.
